# Prevalence of COVID-19 vaccine hesitancy in students: A global systematic review

**DOI:** 10.12688/f1000research.122815.1

**Published:** 2022-08-12

**Authors:** Debendra Nath Roy, Md. Mohabbot Hossen, Mohitosh Biswas, Ekramul Islam, Md.Shah Azam

**Affiliations:** 1 Pharmacy department, Jashore University of Science and Technology, Jashore, 7408, Bangladesh; 2 Institute of Education and Research, University of Rajshahi, Rajshahi, 6205, Bangladesh; 3 Pharmacy discipline, Khulna University, Khulna, Bangladesh; 4 Pharmacy department, University of Rajshahi, Rajshahi, 6205, Bangladesh; 5 Marketing department, University of Rajshahi, Rajshahi, Bangladesh; 6 Office of the Vice chancellor, Rabindra University, Shahjadpur, Bangladesh

**Keywords:** COVID-19, vaccine hesitancy, acceptance, students, global

## Abstract

**Background:** Examining the prevalence of coronavirus disease (COVID-19) vaccine hesitancy and understanding what motivates students to accept or reject a newly promoted vaccine has the potential in preventing rapid spread of infection and optimizing country-wide mass vaccination programs. This systematic review aimed to examine global COVID-19 vaccine hesitancy among students, and to identify an up-to-date and concise assessment of most common factors influencing vaccine acceptance and hesitancy around the world.

**Methods:** A systematic search of peer-reviewed literatures indexed in reputable databases was performed. After obtaining the results via screening using PRISMA flow diagram, a total of 35 articles met the inclusion criteria and formed the basic structure of the study objectives.

**Results:** The results revealed that, the worldwide pooled COVID-19 vaccine hesitancy rate was (x̅%)=29.8% (95% CI 23.37–36.23) among students. According to the country count assessment, the pooled vaccine hesitancy has been found to be ups and downs across the countries around the world such as  (x̅%)=32% (95% CI 20.04–43.97) in Asia, (x̅%)=(28.11%, 95% CI 18.83–37.40) in the United States, (x̅%)=15.59% (95% CI 8.23–22.95) in Europe, (x̅%)=55.93% (95% CI 40.31–71.55) in Africa, (x̅%)=20.4% in North America, and (x̅%)=22.5% in multi-ethnic areas in the reported student’s COVID-19 vaccine hesitancy. In total, 10 key factors were identified. “Side effect” 45.41% (95% CI 29.68–61.14), “safety” 42.27% (95% CI 27.50–57.04), and “trust” 44.95%, (95% CI 26.51–63.39) were the overarching concerns in making student's vaccination decisions.

**Conclusions:** The prevalence of COVID-19 vaccine hesitancy varied among the students; however, vaccine acceptance or refusal relies on several socio-psychological, societal, and vaccine related factors. This study helps the vaccine policy-makers and health stakeholders gain a better understanding of COVID-19 vaccination drive and design the vaccine promotion strategies. Health educational interventions could be the most preferred approach to improve student’s adherence and knowledge about the COVID-19 vaccination consequences.

## Introduction

The morbidity and mortality caused by coronavirus disease 2019 (COVID-19) has led to an unusual health burden across the countries and recognized as a global health warning, and it is not over yet. The World Health Organization (WHO) and public health expertise suggested non-pharmaceutical health measures and non-therapeutic managements for reducing rapid contamination of the novel coronavirus. Alongside, an unprecedented effort from the global scientific community has been paid to discover a new vaccine quickly because vaccines are the most promising and cost-effective health intervention that mitigates the viral infection. Noticeably, mass vaccination has proven its applicability in gradual silencing of pandemic and epidemic since last five decades. ^1^ The Centers for Disease Control and Prevention (CDC) has declared vaccination as one of the ten public health achievements. ^2^ However, the optimization of country-wide vaccination coverage largely depends on the vaccine acceptability among various population subgroups, particularly in students who are more vulnerable due to their active lifestyle and perception of invulnerability.

Despite significant immunization advances in the 21 ^st^ century around the world, there are still significant obstacles to COVID-19 vaccine in the vaccine- based intervention worldwide and one of which is hesitancy or low vaccine acceptance intention. Vaccine hesitancy is characterized by delay in accepting, hesitation, or rejection of vaccine despite the vaccination services being available. ^3^ More specifically, vaccine hesitancy is expressed in “5C” sequences point to confidence, complacency, convenience, communication, and context. ^4^ Accordingly, the World Health Organization declared the vaccine hesitancy as one of the top ten therapeutic challenges. ^5^ Even prior to the pandemic vaccine hesitancy due to social and behavioral influences was identified as health threat ^5^ and this concern is growing for COVID-19 vaccination because vaccine acceptance and hesitancy remarkably varied among various population sub-groups, with substantial regional variability. ^6^

Worldwide, the low vaccine uptake intention or hesitancy towards a particular vaccine has been recognized the most common phenomenon among the student groups in previous vaccination programs such as for influenza vaccination, ^7^^–^^11^ human papilloma virus (HPV) vaccination, ^12^^–^^16^ hepatitis B vaccinations, ^17^^,^^18^ pertussis vaccinations, ^19^ HIV vaccinations, ^20^ measles vaccinations, ^21^ and now COVID-19 vaccinations. ^22^

In context of current COVID-19 pandemic, most of the review studies on vaccine hesitancy were conducted by analyzing the general population sample ^23^^–^^29^ and few emphasized the regional locations. ^30^^–^^32^ Although a study attempted to illustrate the COVID-19 vaccine hesitancy among health care student trainees; ^33^ there is lack of evidence that concentrated to assess COVID-19 vaccine hesitancy among the students globally. Hence, this systematic review aimed to examine global COVID-19 vaccine hesitancy among students, and to identify an up-to-date and concise assessment of most common factors influencing vaccine acceptance and hesitancy around the world.

## Methods

To fine-tune the study objectives, we investigated the popular peer-reviewed databases for summarizing COVID-19 vaccine hesitancy among students. The Preferred Reporting Items for Systematic Reviews and Meta Analyses-(PRISMA) 2020 statement ^34^ flow diagram was employed for screening procedure of databases as well as for the course of literatures selections. Four bibliographic databases (Pub Med, Embase, Science Direct and Google Scholar) were searched to retrieve studies related to COVID-19 vaccine hesitancy and acceptance among students.

The literature search inclusion criteria were the followings: 1) peer-reviewed articles published from four major databases; 2) quantitative survey studies involving student groups as sample population; 3) address the scope and principal aim of the study; 4) original research focused COVID-19 vaccine hesitancy; and 5) English language used in the publication. On the other hand, we track the following exclusion criteria: 1) unpublished manuscripts; 2) publication with lack of required original data; 3) students were not the sample population; 4) articles focused non-COVID-19 vaccine hesitancy; 5) publications other than original research; and 6) publication language was not English. The search period for the review spanned between November 2021 and December 2021.

The permission to conduct this review was obtained from “Ethical Review Committee” (IRC), Faculty of Biological Science and Technology, Jashore University of Science and Technology, Bangladesh. Since no clinical intervention was applied to the subject, the study was not required for ethical approval, although we informed the review matter to the IRC; however considering all issues the IRC opined not to require further approval.

The review protocol sets out the methods to be used in the review and provides an explicit plan. Decisions about the review question, inclusion criteria, search strategy, study selection, data extraction, quality assessment, data synthesis, and plans for dissemination was addressed by the authors collaboratively. The review protocol was further assessed by the IRC, Faculty of Biological Science and Technology, Jashore University of Science and Technology, Bangladesh. Three major themes such as COVID-19 vaccine, vaccine hesitancy, and vaccine acceptance were used to develop search protocol. The key predictive items associated with vaccine hesitancy was conceptualized around COVID-19 vaccines in global perspectives. ^6^ The search items we used in this study were adopted from the theory analysis of prior systematic analysis that evaluated non-COVID-19 vaccine hesitancy around the world ^35^^–^^40^ The literature search for peer-reviewed articles was conducted by using the following keywords: (“novel coronavirus” OR “coronavirus 2019” OR “COVID 2019” OR “COVID19” OR “COVID-19” OR “SARS-CoV-2” OR “HCoV-19” OR “2019-nCoV” OR “severe acute respiratory syndrome corona virus 2”) AND (vaccine * OR immunization) AND (hesitancy * OR reluctance * OR acceptance) AND (student * OR educational sector). The Cochrane collaboration’s review team was formed for assessing the risk of biases and reports the assessment protocol that ensure the process become more accurate. The team attempted to identify, appraise, and synthesize all the empirical evidence that met pre-specified eligibility criteria. The first three independent reviewers conducted preliminary pilot study for screening of first 55 articles based on the titles and abstracts. The same reviewers independently performed screening of the titles and abstracts of all retrieved articles from selected databases. The potentially relevant articles were evaluated for full-text analysis prior to inclusion in the synthesis process. Disagreements raised in inclusion phase of review process were critically evaluated to attain consent. The fourth and fifth reviewer acted as an independent mediator for such disagreements which could not be resolved between first two reviewers. The critical appraisal of study outcomes was evaluated and the expected outcome measures of the study were COVID-19 vaccine hesitant population, associated factors, and the number of population identify the factors.

Microsoft excel data collection sheet was prepared by two authors. The sheet was critically evaluated, reviewed, and approved by third and fourth reviewer to chart the data from included articles. Qualitative method was employed to synthesize the study outcomes. The data were extracted by two authors independently while the third and fourth author reviewed the synthesized data placed in excel sheet. The filled data sheet comprised the key information included author (s) name, study type, study title, year of publication, sample size, population characteristics, study design, and the analytical approach used in the respective studies. Descriptive statistics described percentage and weighted frequencies the study samples. Mean (x̅), standard deviation (SD), and standard error (SE) were calculated to estimate 95% confidence interval (CI).

## Results

### Search Results

**Figure 1.**
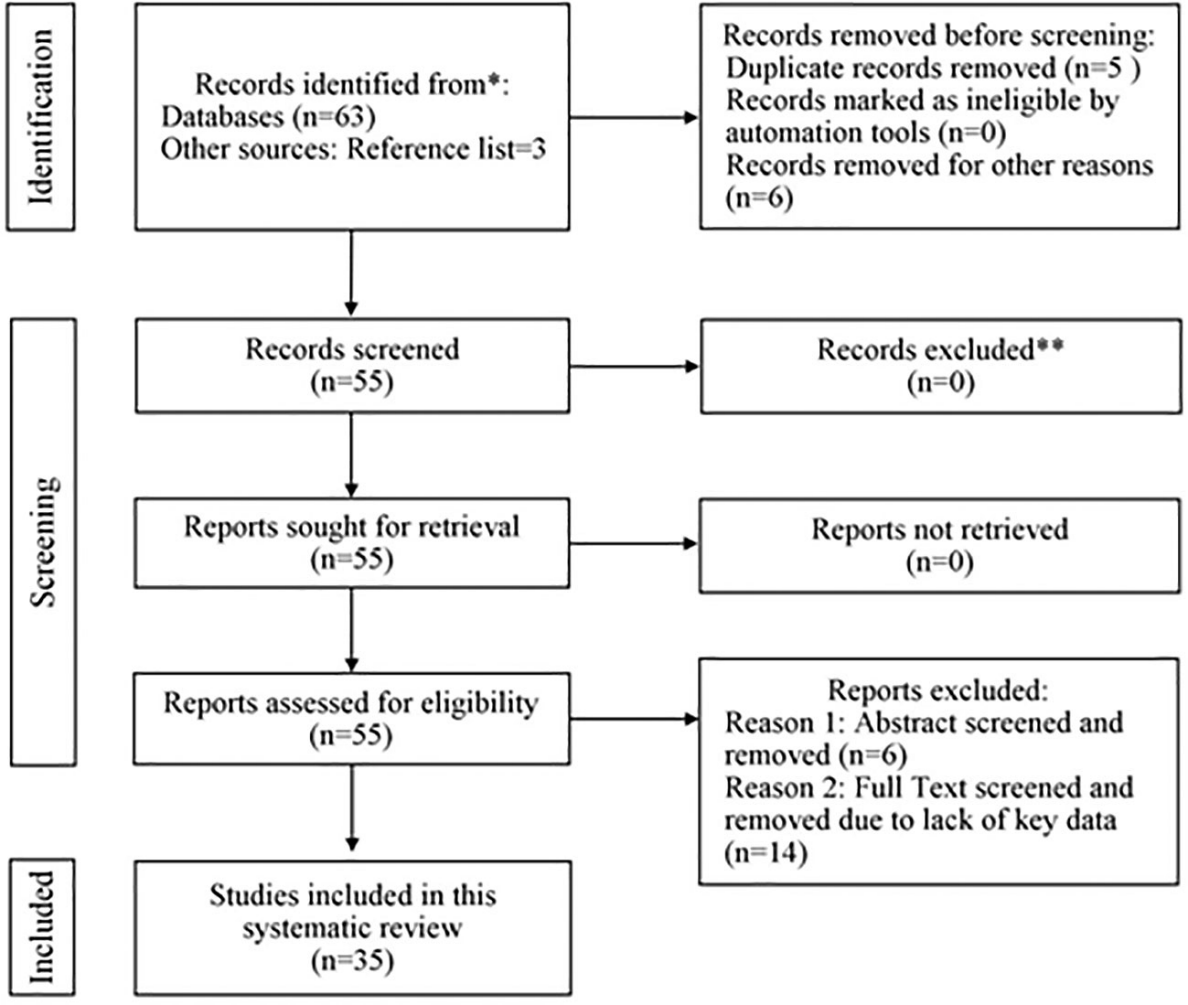
PRISMA-based flow diagram of study selection process for new systematic reviews.

The initial search results retrieved 63 studies from the selected databases. Additionally 3 survey articles were identified from reference lists included in the review process. The search results also included letters, commentary, viewpoints, and conferences that needed to exclude from the review. The review process is shown in Figure 1. In the initial phase and before screening 4 duplicate articles, 3 commentaries, 2 letters, and 2 viewpoints were identified and took away from the procedure while 55 articles were screened. After careful screening the abstract, 6 articles were removed at the eligibility assessment step. The remaining 49 articles were analyzed for full-text; however, to comply with the study objectives the independent reviewer excluded 14 articles because these articles lack the required data. Finally, 35 peer-reviewed articles were selected to include in this study for rationalizing the study objectives. A collaborative editorial team led by the most senior author assesses the risk of associated biases which may occur in the included studies.

### Characteristics of included studies and population

Most of the studies included in this systematic review were carried out when the COVID-19 vaccination has started in the respective country context. The study reduced ambiguity in sample population selection and considered the risk of introducing spectrum bias when selecting study population. The highest number of the studies ( *n*=9) that included in this study were carried out in the USA. From China, 6 studies were included; 3 studies were conducted in Italy, 1 study in France, India, Uganda, Israel, Egypt, Zambia, Poland, Kuwait, Jordan, Czech Republic, Lebanon, Kazakhstan, Nigeria, Saudi Arabia, Bangladesh, Canada, and Romania. One included study represented 22 country populations in multi-ethnicity. The participants were under-graduate and postgraduate students of diverse educational institutes and branches worldwide. Among these student diversities, 10 studies were conducted on university students, 9 studies were conducted on medical students, 7 studies were college students, 2 studies were dental students, 2 studies were nursing students and health care students of each, 1 study was carried out in combined sample of medical and nursing students, 1 multi-ethnic study represented combination of medical and dental students, 1 study conducted among pharmacy students and another 1 study was conducted among the international college students. The collaborative team evaluated individual synthesis for assessing the risk of reporting bias raised from missing values.

### Vaccine hesitancy among students

The overall vaccine hesitancy rate synthesized from eligible 35 articles ^41^^–^^75^ is shown in Table 1. The percentage mean value of the hesitant students was (x̅ %)=29.80 (95% CI 23.37–36.23) and the mean respondents value was (x̅)=341.81 (95% CI 217.76–465.86) while the total population mean was (X)= 1290.58. The highest value of the percent hesitant was reported (xh)=75.6 (95% CI 73–78) and the lowest value reported 3 (95% CI 1–5) among the studied articles.

**Table 1.** The global COVID-19 vaccine hesitancy in students.

[Study count]	Mean total populations (X̅)	Mean respondents (x̅) (95% CI)	Hesitant % (highest, (xh)) (95% CI)	Hesitant % (lowest xl) (95% CI)	Mean hesitant respondents% (x̅ %) (95% CI)
[41-75]	1290.58	341.81 (217.76–465.86)	75.6 (73–78)	3 (1–5)	29.80 (23.37–36.23)

Table 2 summarizes and describes the mode of distribution frequency of hesitancy rate around the world. In Asian countries, we analyzed 13 articles (n=13) in which the percentage mean value of the hesitant students was (x̅%)=32 (95% CI 20.04-43.97) and the mean respondents value was (x̅)=347.31 (95% CI 203.81–490.81) while the total population mean was (X)= 1288.77. The highest value of the percent hesitant was reported (xh)=75.6 (95% CI 73–78) and the lowest value was=(xl) 9.6 (95% CI 5.6–13.6) in Asian countries. The synthesized results obtained in the United States (n=9; x̅%=28.11, 95% CI 18.83-37.40;x̅=136.5, 95% CI 63.54–209.46;xh%= 47.5,95% CI 63.54–209.46; xl%=3, 95% CI 1–5 and X̅=511.3), in Europe (n=7; x̅%=15.59, 95% CI 8.23–22.95; x̅=413.29, 95% CI 216–824.42; xh%= 29, 95% CI 28–30; xl%=5.27, 95% CI 3–7; X̅=1883.86), in Africa (n=4; x̅%=55.93, 95% CI 40.31–71.55; x̅=444.75, 95% CI 85.12–804.38; xh%= 75, 95% CI 70–80; xl%=40, 95% CI 35–45; X̅=874.75), in North America (n=1; x̅%=20.4; x̅=259; xh%= 20.4; xl%=20.4; X̅=1269) and, in multi-ethnic areas (n=1; x̅%=22.5; x̅=1494; xh%= 22.5; xl%=22.5; X̅=6639).

**Table 2.** The country specific COVID-19 vaccine hesitancy in student cohorts.

Ethnicity	Study [count]	Mean total populations (X̅)	Mean hesitant respondents (x̅) (95% CI)	Hesitant % (highest, xh) (95% CI)	Hesitant % (lowest, xl) (95% CI)	Mean hesitant respondents% (x̅ %) (95% CI)
Asia	[41-53]	1288.77	347.31 (203.81–490.81)	75.6 (73–78)	9.6 (5.6–13.6)	32.00 (20.04–43.97)
The USA	[54-62]	511.3	136.5 (63.54–209.46)	47.5 (41.5–54)	3 (1–5)	28.11 (18.83–37.40)
Europe	[63-69]	1883.86	413.29 (2.16–824.42)	29 (28–30)	5.27 (3–7)	15.59 (8.23–22.95)
Africa	[70-73]	874.75	444.75 (85.12–804.38)	75 (70–80)	40 (35–45)	55.93 (40.31–71.55)
North America	[74]	1269	259 ─	20.4 ─	20.4 ─	20.4 ─
Multi-ethnic	[75]	6639	1494 ─	22.5 ─	22.5 ─	22.5 ─

### Factors associated with vaccine acceptance and hesitancy

The most frequently identified factors in vaccination decision are illustrated in Table 3. In total 10 potential factors were identified from our studied articles. Among these key factors “side effect” was identified from the highest count in 15 articles (n=15) in which the percentage mean value of the student respondents was (x̅%)=45.41 (95% CI 29.68–61.14), the mean respondents was (x̅)= 623.87 (95% CI 153.16–1094.58), and the total population mean was (X̅)= 1484.13. The highest value represented side effect reported (ch%)=96.8 (95% CI 96.3–97.7) while the lowest was (cl%)=1.15 (95% CI 1.15–1.95). The second highest count (n=13) was recognized for “safety” (n=13; x̅%=42.27 95% CI 27.50–57.04); x̅=451.92 95% CI 122.37–781.48; X̅=1285.62; ch%=84.3 95% CI 80.3–88.3; cl%=4.3 95% CI 3.8–4.8), followed by “trust” (n=9; x̅%=44.95, 95% CI 26.51–63.39; x̅=414.44, 95% CI -12.51–841.39; X̅=1189; ch%= 89, 95% CI 85-93–cl%=10.6, 95% CI 8.6–12.6), “information sufficiency” (n=6; x̅%=47.05 95% CI 25.45–68.65; āx̅=715.5, 95% CI 9.93–1421.07; X̅=1672.67; ch%= 77.3, 95% CI 72.3–82.3; cl%=16, 95% CI 11–21), “effectiveness” (n=6; x̅%=46.22, 95% CI 19.26–73.19; x̅=708.17, 95% CI 3.81–1412.53; X̅=991.17; ch%= 93.2 95% CI 92–94; cl%=10.2, 95% CI 7.2–13.2), “efficacy” (n=4; x̅%=24.97 95% CI -12.36–62.30; x̅=179.67, 95% CI -16.00–375.34; āX̅=1585.67; ch%=62.7 95% CI 58.7–66.7; cl%=1.6 95% CI 1.2–2), “vaccine mandate” (n=3; x̅%=52.7 95% CI 9.13–96.27; x̅=124.67 95% CI 16.13–233.21; X̅=217.33; ch%= 85, 95% CI 80–90; cl%=10.1, 95% CI 5.1–15.1), “social influence” (n=2; x̅%=33.98 95% CI -31.53 – 99.49; x̅=92 95% CI -55– 239; X̅=1668.5; ch%= 67.4, 95% CI 62.4–72.4; cl%=0.55, 95% CI 0.2 – 0.8), “conspiracy beliefs” (n=2; x̅%=17.15 95% CI 14.31–19.99; x̅=258 95% CI 156.08 – 359.92; X̅=1538.5; ch%= 18.6, 95% CI 16.6–20.6; cl%=15.7, 95% CI 13.7–17.7), and “religiosity” (n=1; x̅%=12.3; x̅=817; X̅=6639).

**Table 3.** Factors influencing COVID-19 vaccine acceptance and hesitancy in student cohorts.

Factors	No of study	Mean total populations (X̅)	Mean respondents (x̅) (95% CI)	Concern % (highest, ch) (95% CI)	Concern % (lowest, cl) (95% CI)	Mean respondents% (x̅ %) (95% CI)
Side effect	15	1484.13	623.87 (153.16–1094.58)	96.8 (96.3–97.7)	1.55 (1.15–1.95)	45.41 (29.68–61.14)
Safety	13	1285.62	451.92 (122.37–781.48)	84.3 (80.3–88.3)	4.3 (3.8–4.8)	42.27 (27.50–57.04)
Trust	9	1189	414.44 (-12.51–841.39)	89 (85–93)	10.6 (8.6–12.6)	44.95 (26.51–63.39)
Information sufficiency	6	1672.67	715.5 (9.93–1421.07)	77.3 (72.3–82.3)	16 (11–21)	47.05 (25.45–68.65)
Effectiveness	6	991.17	708.17 (3.81–1412.53)	93.2 (92–94)	10.2 (7.2–13.2)	46.22 (19.26–73.19)
Efficacy	4	1585.67	179.67 (-16.00–375.34)	62.7 (58.7–66.7)	1.6 (1.2–2)	24.97 (-12.36–62.30)
Vaccine mandate	3	217.33	124.67 (16.13–233.21)	85 (80–-90)	10.1 (5.1–15.1)	52.7 (9.13–96.27)
Social influence	2	1668.5	92 (-55–239)	67.4 (62.4 – 72.4)	0.55 (0.2 – 0.8)	33.98 (-31.53–99.49)
Conspiracy beliefs	2	1538.5	258 (156.08– 359.92)	18.6 (16.6–20.6)	15.7 (13.7– 17.7)	17.15 (14.31–19.99)
Religiosity	1	6639	817			12.3

**Figure 2.**
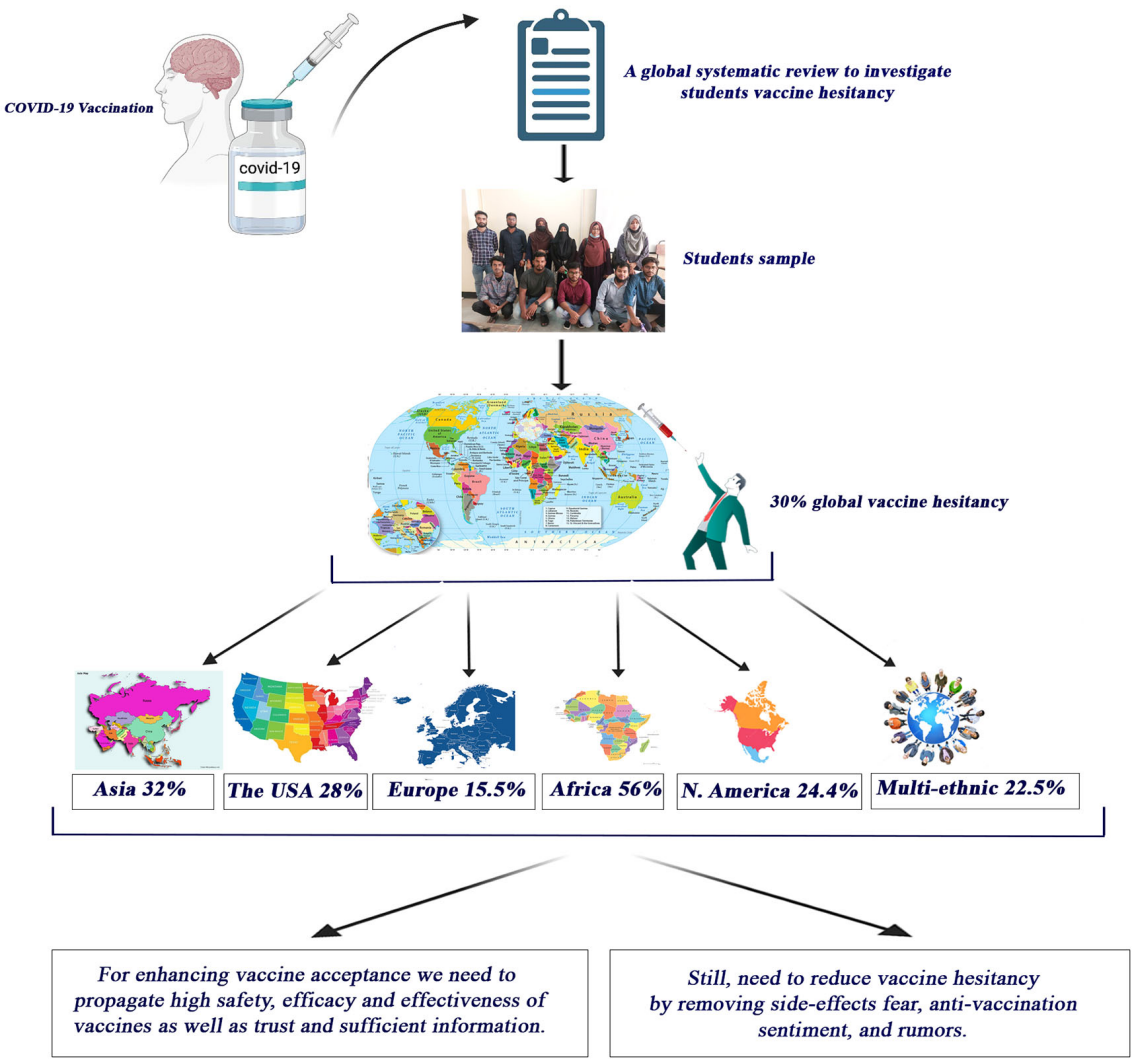
A graphical view of the overall study outcomes in the included studies.

In Figure 2, we represented a graphical view of the overall study outcomes that we have extracted and summarized from included eligible articles.

## Discussion

The COVID-19 pandemic not only has destroyed the economic development, health systems, and transport but also the education system was brutally affected worldwide. Despite a widespread discussion about the effect of pandemic on economy and health system, the catastrophic impact of lethal coronavirus on education system has yet to draw the attention of the world’s community to a large extent. Most developed nations have succeeded in overcoming the disastrous impact of this virus through the online transformation of their traditional education system and rapid vaccination coverage to the students because the ratio of vaccine coverage is comparatively higher in developed countries than that of developing nation. ^76^ Vaccine hesitancy or vaccine refusal is not an old phenomenon; rather, it is dynamic and heterogeneous concept sharply regulated by multi-faceted events of socio-psychological, societal, and behavioral characteristics. ^35^ Ensuring the equitable vaccine coverage among different population groups is facing challenges because public perceptions on vaccination may alter over time and disease backdrop. ^77^ Even for national immunization programs, the psychological behaviors explaining vaccine uptake intention is almost similar as in pandemic crisis. ^78^ Acceptability of a newly promoted vaccine is the prime indicator of a successful vaccination drive while at the same time vaccine may economically costly and the equal distribution would time consuming process. ^79^ As such, synthesizing and summarizing the global COVID-19 vaccine hesitancy in student groups would be an effective step to formulate the strategies that boost mass vaccination programs by reducing COVID-19 vaccine hesitancy. This study thus investigated global COVID-19 vaccine hesitancy in students and explored the potential factors associated with it. During the early days of vaccination, a smear campaign was set out to embarrass the health policy makers and many groups intentionally aired different propaganda about vaccine origin and vaccines data. Consequently, globally, a significant portion of population in different geographical locations remained confused about whether they should accept or reject COVID-19 vaccine. Recently a systematic review reported that, 40% sample population had the hesitancy or refusal intention in accepting a COVID-19 vaccine. ^80^ In our study, the pooled COVID-19 vaccination hesitancy rate was identified 30% among students globally. Since the selected study population was students, who are resourceful in accessing the updated vaccine information. Therefore, a relatively low hesitancy rate was observed in student’s cohort compared to the general population in prior study. Since 2014, hesitancy towards a particular vaccine has increased exponentially in more than 90% countries of the world. ^81^ The current study findings indicated that, the overwhelming majority of the hesitant students need to integrate into mass vaccination process, otherwise global attempts to provide free vaccines may not be effective in preventing COVID-19 transmission and morbidity.

Several factors potentially contributed to the vaccination decision and plays a role in individual’s behavior to refuse, delay, or accept vaccines reported in previous measles and pertussis outbreak ^82^ and now for COVID-19 vaccination consequences ^6^. This study deduced vaccine safety, side effects, and trust were the most common factors responsible for student’s COVID-19 vaccine acceptance and hesitancy. Several scientific studies explained previous vaccination progress reported same factors as the vaccine predictive concerns. For example, a comprehensive review synthesized data from 2,791 studies published between 1990 and 2019 concluded that, safety was the principal predictor of vaccine refusal alongside with disease severity, culture, and contextual determinants. ^83^ In the same manner, side effects and safety were the primary considerations in vaccine receiving decision by the general people and health care professionals retrieved from 1,187 articles focused on global HPV and flu vaccinations. ^84^ Similarly, Karafillakis and Larson (2017) argued that, among others vaccine safety and efficacy were the highest concerns in making vaccination decision since 2004 to 2014 among the English, French, and Spanish nations. ^85^

Trust plays a key role in modifying the public behaviors toward vaccine apprehension because restoring public trust would lead to COVID-19 vaccine confidence. ^86^ When the public trusted that, the prospective vaccines would be safe and effective after inoculation vaccine confidence would be built. This growing confidence was the greatest forecaster in making vaccine uptake decision by the public. ^87^ As a result, trust has been recognized as one of the most important predictors of vaccine acceptance in low and middle income countries. ^88^ Alternatively, distrust of and misinformation about the vaccines, and the government agencies regarding the vaccination process significantly reduced the vaccine acceptance rate. ^89^ Hence, building public trust and confidence in health systems would be the key solutions for reducing COVID-19 vaccine hesitancy. It is also important that, evidence-based information need to be provided by the independent expert groups for tracking and tackling of fake news about the vaccines already circulated to the general people.

This study addressed some limitations. The foremost limitation of this study was inadequate sample size, because we synthesized limited number of scholarly articles included in analytical estimation purpose. A lot of peer-reviewed articles have been published in the current COVID-19 vaccine context. As a result, there is a possibility for those articles in which the student participants might have been more accepting or hesitant than our included studies. Secondly, most of the analyzed articles were cross-sectional types, thus provided snapshots of hesitancy status in each country. Actually, it is absolutely challenging to predict in-spot vaccine perceptions among people because vaccine apprehension may depend on disease backdrop and it can alter over time. ^77^ Thirdly, we have documented few selective factors of COVID-19 vaccine acceptance and hesitancy in students; however, this may differ from behavioral context. Due to the disease severity, perceived health risk, pandemic backdrop, and approval of new COVID-19 vaccines the possibility of temporal changes in factors associated with COVID-19 vaccinations would take place. Finally, there are some additional key factors including rumors ^90^^,^^91^ observed in Asian continents were truly unidentified in this study.

Given the prevalence of vaccine hesitancy and potential influential factors of COVID-19 vaccinations, the educational policy makers should develop strategies that compliance with the adherence, attitude, and knowledge about vaccination consequences among the students group. This study acts as scientific evidence for initiating further predictive studies of COVID-19 vaccine acceptance and hesitancy among students by examining the association between vaccine acceptance and other explanatory variables. Since educational contents aimed to improve infectious disease awareness among students would be beneficial, ^92^ hence the advancement of effective health education in a multi-disciplinary approach would be imperative to emphasize personal relevance of the disease and to improve vaccine related knowledge among the students. The authors believe that, the study findings largely benefits the health policy makers, stakeholders, and the vaccine promoters in different parts of the world to design an evidence-based vaccine promotion strategy seeking to influence the vaccination policy implication in pandemic and post pandemic era.

## Conclusions

Since COVID-19 vaccine availability does not guarantee uptake, so examining COVID-19 vaccine hesitancy among students and identifying the factors associated with vaccine acceptance and hesitancy is a fundamental task that must be undertaken to guarantee an effective immunization plan. This study investigated global COVID-19 vaccine hesitancy among the students and concluded that the vaccine hesitancy was higher in African countries followed by Asia, the United States and Europe. Although the prevalence of COVID-19 vaccine hesitancy varied among the higher education students globally; vaccine acceptance relies on several common factors related to socio-psychological and the vaccine itself. In this study, we identified 10 potential concerns related to vaccine uptake and refusal intention in students, among which side effect, safety, trust, and information sufficiency were the most prominent concerns of COVID-19 vaccination decision among students. Hence, the scientific community must ensure the safety confirmation, side-effect free remedy, rapid response against disease; provide long-term therapeutic benefit, and acquisition of required immunity to encounter the perceived challenges in successful vaccination programs. In addition, the manufacturers need to produce adequate vaccine doses and distributed vaccines equally across the countries. Public perceptions are likely to be changed as more vaccine related safety and efficacy data become largely available and convey the information to people through effective communication and trustworthy approach. COVID-19 vaccine information regarding side effects, safety, and efficacy as well as the communicative roles of the media is essential for improving vaccine trust among the students. Application of useful communication channels and educational interventions would contribute to remove anti-vaccination sentiments and rumors thereby enhancing vaccine uptake willingness among students. Effective policy directions for pandemic management and vaccination consequences in the academic curricula would shape and influence education community to enhance student’s health engagement in infectious disease awareness and vaccine readiness. Therefore, addressing COVID-19 vaccine hesitancy among students and enlisting the factors associated with vaccine acceptance and hesitancy is a fundamental assignment that must be undertaken to guarantee an immunization plan throughout the country. The study findings thus help the researchers, policymakers, and administrators gain a better understanding of vaccination drive among students and call for further implementation of multi-disciplinary educational intervention within the academic curricula.

## Data availability

### Underlying data

No data are associated with this article.

### Reporting guidelines

Figshare: PRISMA checklist for ‘Prevalence of COVID-19 vaccine hesitancy in students: A global systematic review’. https://doi.org/10.6084/m9.figshare.20366712. ^93^

Data are available under the terms of the Creative Commons Attribution 4.0 International license (CC-BY 4.0).

## Consent

The authors confirm that, we have obtained signed consent from the participants included in the graphical presentation ( Figure 2) to exploit images in the article. Also, the graphical presentation contained no reproduced copyrighted materials from other sources while the image was self-generated by the authors.
