# Supplementary material for: Prevalence of COVID-19 vaccine hesitancy in students: A global systematic review
Source: F1000Res. 2022 Aug 12;11:928. [Version 1] doi: 10.12688/f1000research.122815.1 (PMC11406128; doi:10.12688/f1000research.122815.1)
Supplement: Peer review history

# Reviewer report: Reviewer response for version 1

**DOI:** 10.5256/f1000research.134854.r189184

**Published:** 2024-09-16

**Authors:** Lakshit Jain

**Affiliations:** 1 Department of Psychiatry, University of Connecticut, Storrs, Connecticut, USA; 2 Connecticut Valley Hospital, Middletown, Connecticut, USA

As someone for whom English is not their primary language, this reviewer understands the struggle in writing in a different language. 

 The article uses excessively ornate language with too many adjectives. Such language is more suited for an opinion piece or an editorial, not for a review article. 

 At certain places in the article Authors talk about specific countries, but at other places they transition to continents. Please adhere to one set of terminology. 

 The Authors write "screening using PRISMA flow diagrams" on multiple occasions. This is incorrect. One follows the PRISMA guidelines and uses the diagram to describe to the reader how the authors adhered to the guidelines.

Are the rationale for, and objectives of, the Systematic Review clearly stated?

Yes

Is the statistical analysis and its interpretation appropriate?

Yes

If this is a Living Systematic Review, is the ‘living’ method appropriate and is the search schedule clearly defined and justified? (‘Living Systematic Review’ or a variation of this term should be included in the title.)

Yes

Are sufficient details of the methods and analysis provided to allow replication by others?

Yes

Are the conclusions drawn adequately supported by the results presented in the review?

Yes

Reviewer Expertise:

Psychiatry, Addiction Psychiatry

I confirm that I have read this submission and believe that I have an appropriate level of expertise to confirm that it is of an acceptable scientific standard, however I have significant reservations, as outlined above.

# Reviewer report: Reviewer response for version 1

**DOI:** 10.5256/f1000research.134854.r189170

**Published:** 2023-11-28

**Authors:** Jomell M Santiago

**Affiliations:** 1 College of Education, Nueva Ecija University of Science and Technology, Cabanatuan City, Central Luzon, Philippines

The introduction should contain literature about vaccine hesitancy in a global or worldwide approach. As written in the introduction, only one stanza discusses this section which is not sufficient. Thus, it is recommended to increase the number of literature that will address or discuss this part since the center of this paper is about vaccine hesitancy worldwide. 

 The methods were fully explained all the sections were stated very well.

 In the result, there were 63 studies from the selected databases and 35 articles chosen. Is the number of articles/sources enough to be the basis of the study? It is recommended (if possible) to add articles that will somehow cover the majority of the countries to establish vaccine hesitancy worldwide. For the discussion part, all the sections that must be explained were stated and discussed very well.

 The conclusion should be reduced or stated in the very least statements as much as possible. 

 Use proper formatting/style in writing the references. Include its doi or the link where it is derived.

Are the rationale for, and objectives of, the Systematic Review clearly stated?

Yes

Is the statistical analysis and its interpretation appropriate?

Not applicable

Are sufficient details of the methods and analysis provided to allow replication by others?

Yes

Are the conclusions drawn adequately supported by the results presented in the review?

Yes

Reviewer Expertise:

Education, biological science, social science, science education

I confirm that I have read this submission and believe that I have an appropriate level of expertise to confirm that it is of an acceptable scientific standard, however I have significant reservations, as outlined above.

# Response

**Published:** 2023-11-28

**Authors:** Debendra Roy

**Affiliations:** Jashore Univer sity of Science and Technology, Bangladesh

Reviewer: The introduction should contain literature about vaccine hesitancy in a global or worldwide approach.

 Author response: Thanks for your valuable comments. We have conducted this study at the early phase of vaccination derive (between November 2021 and December 2021) and primarily focused on the students vaccine hesitancy globally. There was a lack of review on students’ COVID-19 vaccination decision, so we could not include maximum references highlighted COVID-19 vaccine reluctance in the introduction section. To rationalize the study’s outlined objectives, we incorporated many study (review) articles related to other vaccinations types (ref: 12-21) conducted on students perspectives. We strictly denied mentioning the research article as a citation in the introduction phase. I hope these numbers satisfy the study requirement.

 Reviewer: The methods were fully explained all the sections were stated very well.

 Author response: Thanks for your appreciation.

 Reviewer: In the result, there were 63 studies from the selected databases and 35 articles chosen. Is the number of articles/sources enough to be the basis of the study? It is recommended (if possible) to add articles that will somehow cover the majority of the countries.

 Author response: Thanks for your observation. Although we noticed many more relevant article currently; however, we have conducted this study at the early phase of vaccination derive (between November 2021 and December 2021). Limited number of articles published in the topic and this review was the first systematic review on students COVID-19 vaccine decision.  We added a common key word “AND (student * OR educational sector)” with our used keywords during searching the literatures. As a result, we found limited number articles (63) in the search engine. Based on our inclusion criteria we were able to consider 35 articles included in the final analysis. Although there are many articles published recently, however given the study’s originality we would like to remain the same analysis.

 Reviewer: The conclusion should be reduced or stated in the very least statements as much as possible.

 Author response: Thanks for your suggestion. This is a global systematic review on students’ vaccination consequence, so we have incorporated some additional sentences in the conclusion part as a recommendation of the study outcome.  We organized the writing content according to the guideline of the journal.  

 Reviewer: Use proper formatting/style in writing the references. Include its doi or the link where it is derived.

 Author response: Thanks for your suggestion. This journal already published the references. So we believed that the writing met the standard of journal’s referencing style.

# Reviewer report: Reviewer response for version 1

**DOI:** 10.5256/f1000research.134854.r147408

**Published:** 2022-08-30

**Authors:** ASM Monjur Al Hossain

**Affiliations:** 1 Department of Pharmaceutical Technology, Faculty of Pharmacy, University of Dhaka, Dhaka, Bangladesh

1.  
**Relevance of the study: **In the actual context, the paper presents theoretical and practical importance. It is quite interesting the analysis of the global vaccine hesitancy rate and the ten concerns which determine the acceptance or hesitancy towards the Covid-19 vaccines among students globally.
2. **Abstract:** Well organized and concisely stated the review outcomes.
3. **Introduction** – Informative and properly contextualized the current research gap. It summarized relevant research to provide context and to explain the findings to others.
4. **Literature cited**– Adequately cited with appropriate kinds of literature in the current context, hence, no missing or incomplete citations were found. Authors paid appropriate credit to ideas, concepts and data that have been published recently.
5.  
**Methodologically**, I am convinced that the search protocol had drawn strictly. The choice to include key terms is specific and accurately derived has been cited from the existing theory and explained in the protocol development section.
  The shared PRISMA 2020 checklist provided unique information necessary in the manuscript writing and all PRISMA 2020 critical items for a systematic review have been addressed.
  Although it would be good to include a large sample size in data; this review included 35 articles which are acceptable. However, one question can be opened up? If you use any tools to calculate 95% confidence please include? Additionally, you can add an Ethical permission letter as supplementary data.
6.  
**Conclusion **– Strategically reflected the study findings and suggested the recommendation effectively.

Are the rationale for, and objectives of, the Systematic Review clearly stated?

Yes

Is the statistical analysis and its interpretation appropriate?

Yes

Are sufficient details of the methods and analysis provided to allow replication by others?

Yes

Are the conclusions drawn adequately supported by the results presented in the review?

Yes

Reviewer Expertise:

Pharmaceutics, Topical drug delivery, public health, Fungal disease

I confirm that I have read this submission and believe that I have an appropriate level of expertise to confirm that it is of an acceptable scientific standard.
